# Advancing cerebral small vessel disease diagnosis: Integrating quantitative susceptibility mapping with MRI‐based radiomics

**DOI:** 10.1002/hbm.70022

**Published:** 2024-09-10

**Authors:** Zhenyu Cheng, Linfeng Yang, Changhu Liang, Meng Li, Xianglin Li, Yiwen Chen, Pengcheng Liang, Yuanyuan Wang, Xinyue Zhang, Na Wang, Yian Gao, Chaofan Sui, Lingfei Guo

**Affiliations:** ^1^ School of Medical Imaging Binzhou Medical University Yantai Shandong China; ^2^ Department of Radiology Jinan Maternity and Child Care Hospital affiliated to Shandong First Medical University Jinan Shandong China; ^3^ Key Laboratory of Endocrine Glucose & Lipids Metabolism and Brain Aging, Ministry of Education, Department of Radiology Shandong Provincial Hospital Affiliated to Shandong First Medical University Jinan Shandong China; ^4^ Department of Psychiatry and Psychotherapy Jena University Hospital Jena Germany

**Keywords:** cerebral small vessel disease, quantitative susceptibility mapping, radiomics

## Abstract

Cerebral small vessel disease (CSVD) is a neurodegenerative disease with hidden symptoms and difficult to diagnose. The diagnosis mainly depends on clinical symptoms and neuroimaging. Therefore, we explored the potential of combining clinical detection with MRI‐based radiomics features for the diagnosis of CSVD in a large cohort. A total of 118 CSVD patients and 127 healthy controls underwent quantitative susceptibility mapping and 3D‐T1 scans, and all completed multiple cognitive tests. Lasso regression was used to select features, and the radiomics model was constructed based on the regression coefficients of these features. Clinical cognitive and motor tests were added to the model to construct a hybrid model. All models were cross‐validated to analyze the generalization ability of the models. The AUCs of the radiomics and hybrid models in the internal test set were 0.80 and 0.87, respectively. In the validation set, the AUCs were 0.77 and 0.79, respectively. The hybrid model demonstrated higher decision efficiency. The Trail Making Test, which enhances the diagnostic performance of the model, is associated with multiple brain regions, particularly the right cortical nuclei and the right fimbria. The hybrid model based on radiomics features and cognitive tests can achieve quantitative diagnosis of CSVD and improve the diagnostic efficiency. Furthermore, the reduced processing capacity due to atrophy of the right cortical nucleus and right fimbria suggests the importance of these regions in improving the diagnostic accuracy of the model.

## Graphical abstract

The machine learning model based on prospective cohort image features was constructed and improved with clinical indicators, and clinically significant diagnostic criteria for cerebral small vessel disease were determined.

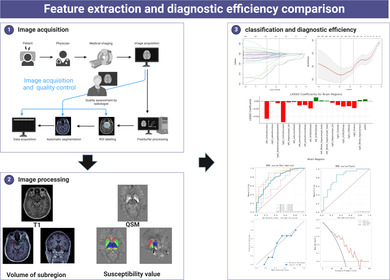

## 1 INTRODUCTION

Cerebral small vessel disease (CSVD) is a neurodegenerative disease characterized by significant age‐related changes, predominantly affecting neurons in specific brain regions. Diagnosis primarily relies on clinical symptoms and neuroimaging. However, early disease diagnosis is challenging due to variable clinical presentations that often overlap with symptoms of other degenerative diseases. The main diagnostic criteria for CSVD include cerebral microbleeds, perivascular spaces, and white matter hyperintensities (Duering et al., 2023). Although these criteria cover a range of CSVD‐related brain damage, diagnosis remains highly dependent on the radiologist's visual assessment. Consequently, there is an urgent need for accurate quantitative diagnostic indices to enhance the precision of imaging diagnoses for CSVD. Amygdala‐hippocampus system is the core structure in the cognitive impairment (CI) of CSVD, where its microstructure damage and disrupted connectivity often result in cognitive decline, especially in CSVD patients (Papma et al., 2014). Numerous previous studies have linked the volumes of the amygdala and hippocampus with CSVD progression, but most of the studies focused on the overall volume, overlooking the significance of changes in subregions that affect cognitive functions in CSVD patients (Perosa et al., 2024). Furthermore, with the deepening of the research on cerebral subcortical iron deposition, the relationship between subcortical iron deposition and neuronal damage has also been confirmed, highlighting neuronal damage as a key characteristic of CI (Lee & Kovacs, 2024). Therefore, the identification of imaging markers that can quantify and visualize iron deposition has become increasingly popular over the years. Quantitative susceptibility mapping (QSM) offers a method for measuring brain iron deposition, yet its diagnostic reliability is limited by age‐related variability in iron levels (Madden & Merenstein, 2023). There is still a lack of theoretical support for diagnosis based on QSM alone. Radiomics is an emerging form of imaging analysis that can address this issue by mining high‐throughput quantitative imaging features. In recent years, radiomics has been increasingly used in the study of disease diagnosis and prognosis, including magnetic resonance imaging (MRI), computed tomography, and positron emission tomography. While its application in CSVD diagnosis remains unexplored, we aim to develop a diagnostic model using radiomics in combined with subregion volume and subcortical nuclear iron deposition characteristics to facilitate early and quantitative CSVD diagnosis.

CI is a significant manifestation of CSVD, characterized by diverse clinical features such as declines in executive functions, attention, and control abilities, and its prevalence increases with age and the severity of the disease (Jia et al., 2020). At present, the specific mechanism of CI remains unclear, but a number of studies have confirmed the crucial role of amygdala and hippocampus volume changes in cognitive decline (Xiao et al., 2023). Additionally, alterations in iron deposition serve as important markers of neuronal damage in the brain (Dusek et al., 2022). We further hypothesized that the combination of subcortical nuclear susceptibility and nuclear subregion volume could provide insights into the relationship between CI and disease progression in CSVD patients.

Our research aims to investigate whether QSM and radiomics could distinguish CSVD patients from healthy controls (HC), and to investigate the mechanisms underlying impaired processing abilities in CSVD patients. The results of this study may enable a breakthrough in the early detection of CSVD, providing new insights into patient management and treatment planning.

## 2 MATERIALS AND METHODS

### 2.1 Participants

**FIGURE 1:**
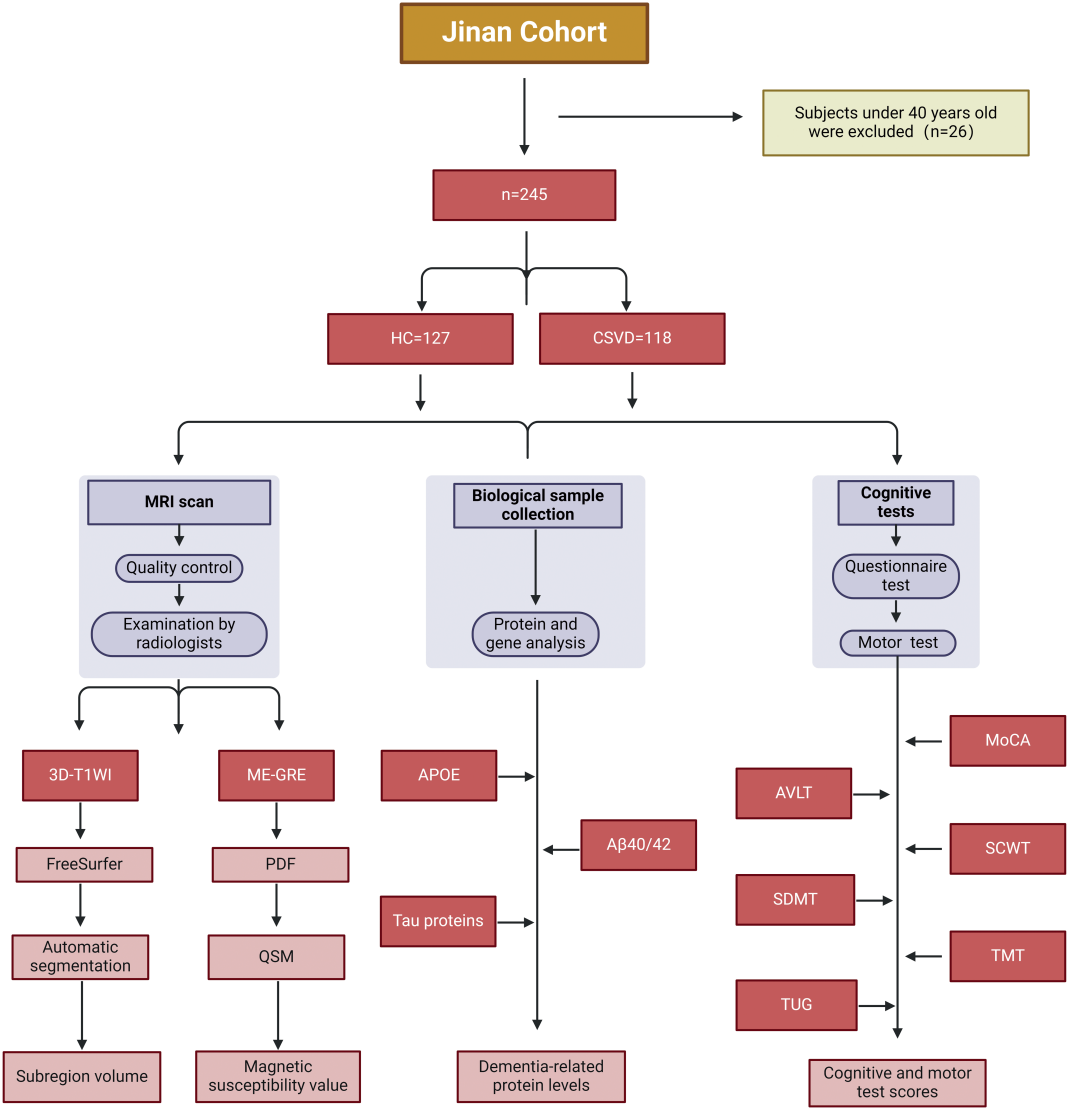
Flowchart of cohort patient selection process and inclusion and exclusion criteria. AVLT, Auditory Verbal Learning Test; ME‐GRE, multiple echo gradient echo; MoCA, Montreal Cognitive Assessment; PDF, Projection onto dipole fields; QSM, quantitative susceptibility mapping; SCWT, Stroop Color‐Word Test; SDMT, Symbol Digit Modalities Test; TMT, Timed Up and Go Test; TMT, Trait‐Motivation‐Test. The TMT we chose is TMT (A + B).

The study had been approved by the Ethics Review Board, and all subjects provided written informed consent. All subjects were from the cohort recruited by Shandong Provincial Hospital Affiliated to Shandong First Medical University. CSVD was diagnosed by Dr. Guo, a radiologist with 20 years of experience in the diagnosis of CSVD, according to the “Neuroimaging standards for research into small vessel disease and its contribution to ageing and neurodegeneration,” combined with clinical cognitive indicators and imaging characteristics (Wardlaw et al., 2013). The secondary diagnosis was completed by Dr. Liang, a senior radiologist with 30 years of experience. The subjects with a common diagnosis of CSVD were determined as the CSVD group, and the subjects with inconsistent diagnosis were excluded. All diagnoses were made independently by clinicians who were completely unaware of the patients until the diagnosis was made. A total of 118 CSVD patients met the inclusion criteria in this study. Inclusion criteria were as follows: (1) Subjects were over 40 years old. (2) Subjects could tolerate MRI scanning. (3) The subjects did not suffer from other diseases that may cause abnormal iron metabolism and atrophy of subcortical nuclei, such as cerebral hemorrhage, cerebral infarction, Alzheimer's disease (AD), and Huntington's disease. (4) Subjects did not receive recent iron supplementation or related proton implants, which may have resulted in inaccurate measurements. (5) The subjects had not taken any psychotropic drugs recently, which could avoid affecting the subsequent cognitive assessment. Exclusion criteria were as follows: (1) poor image quality: 10 subjects were excluded from this study due to poor image quality and inability to perform image post‐processing and (2) incomplete or significantly biased clinical or imaging data: two patients were excluded from the study due to incomplete cognitive assessment (Figure 1).

In addition, we recruited 127 healthy subjects. The healthy subjects were selected according to strict exclusion criteria, and each participant underwent an initial health screening to ensure that they did not have any neurodegenerative disease.

### 2.2 MRI image acquisition and QSM post‐processing

MRI data from all the subjects were acquired with a 3.0‐T MR System (Siemens Healthcare, Erlangen, Germany), which is equipped with a 32‐channel head coil for receiving signals. The anatomical structure images were obtained using the 3D‐T1WI‐MPRAGE (magnetization‐prepared rapid gradient‐echo) sequence to acquire high‐resolution three‐dimensional brain MRI data with the following parameters: repetition time (TR) = 2300 ms; echo time (TE) = 2.3 ms; matrix = 256 × 256; field of view = 24 × 24 cm (Papma et al., 2014); flip angle = 9°; voxel size = 1 × 1 × 1 mm (Perosa et al., 2024). The three‐dimensional multi‐echo gradient‐echo sequence (3D ME‐GRE) parameters were as follows: TR = 50 ms, initial TE = 6.8 ms, TE interval = 4.1 ms, number of echoes = 10, flip angle = 15°, voxel size = 1 × 1 × 2 mm (Perosa et al., 2024), covering the entire brain.

In this study, image analyses were performed using the FreeSurfer image analysis suite (v. 6.0, http://surfer.nmr.mgh.harvard.edu/) implemented by fMRIPrep 20.1.1 (Esteban et al., 2019), followed by the toolbox of segmentation of hippocampal subregions and amygdala (Saygin et al., 2017). The QSM was calculated on the Matlab19 (MathWorks, Natick, MA, USA) platform. After the original ME GRE data were input, the total field was obtained by the nonlinear fitting of ME‐GRE phase. Local fields are calculated by projection to dipole field algorithm (Figure 1). The QSM maps were then computed by means of a morphology‐enabled dipole inversion with automatic uniform cerebrospinal fluid zero reference algorithm (MEDI+0) (Si et al., 2023). Subsequent QSM subcortical nucleus segmentation was performed according to the segmentation pattern of Tian et al. (2020).

After the segmentation process, an experienced neuroradiologist with more than 20 years of experience in neuroradiology performed the visual inspection and screening (Figure 1).

### 2.3 Cognitive assessment

In our study, all participants underwent the Beijing version of the Montreal Cognitive Assessment (MoCA) (www.mocatest.org), an 11‐item examination covering eight cognitive domains including attention and concentration, executive function, memory, language, visual structural skills, abstract thinking, calculation, and orientation (Yu et al., 2012). To provide a rapid screening tool for cognitive dysfunction. In addition, participants completed the Trail Making Test (TMT), which has two parts: TMT‐A to assess cognitive function and TMT‐B to measure executive function. The sum of the time required for the two parts was calculated as TMT, which reflects an individual's combined performance on these cognitive tasks through the time sum. The Auditory Verbal learning test (AVLT) is a neuropsychological test to detect event memory function, which comprehensively assesses the event memory ability of subjects through auditory verbal recall tasks. Symbol‐digit conversion test (SDMT) is a cognitive test method based on digit symbols, which evaluates information processing speed and visual attention by asking subjects to quickly match symbols and digits. The Stroop Color and Word Test (SCWT) is one of the mainstream aptitude tests, which is usually used to assess executive function and attention control to deal with information conflict. All the above cognitive tests were performed by professional neurologists, who were unaware of the disease group and other conditions of the participants, and all the tests were conducted in an independent and undisturbed professional venue (Figure 1).

### 2.4 Feature selection

**FIGURE 2:**
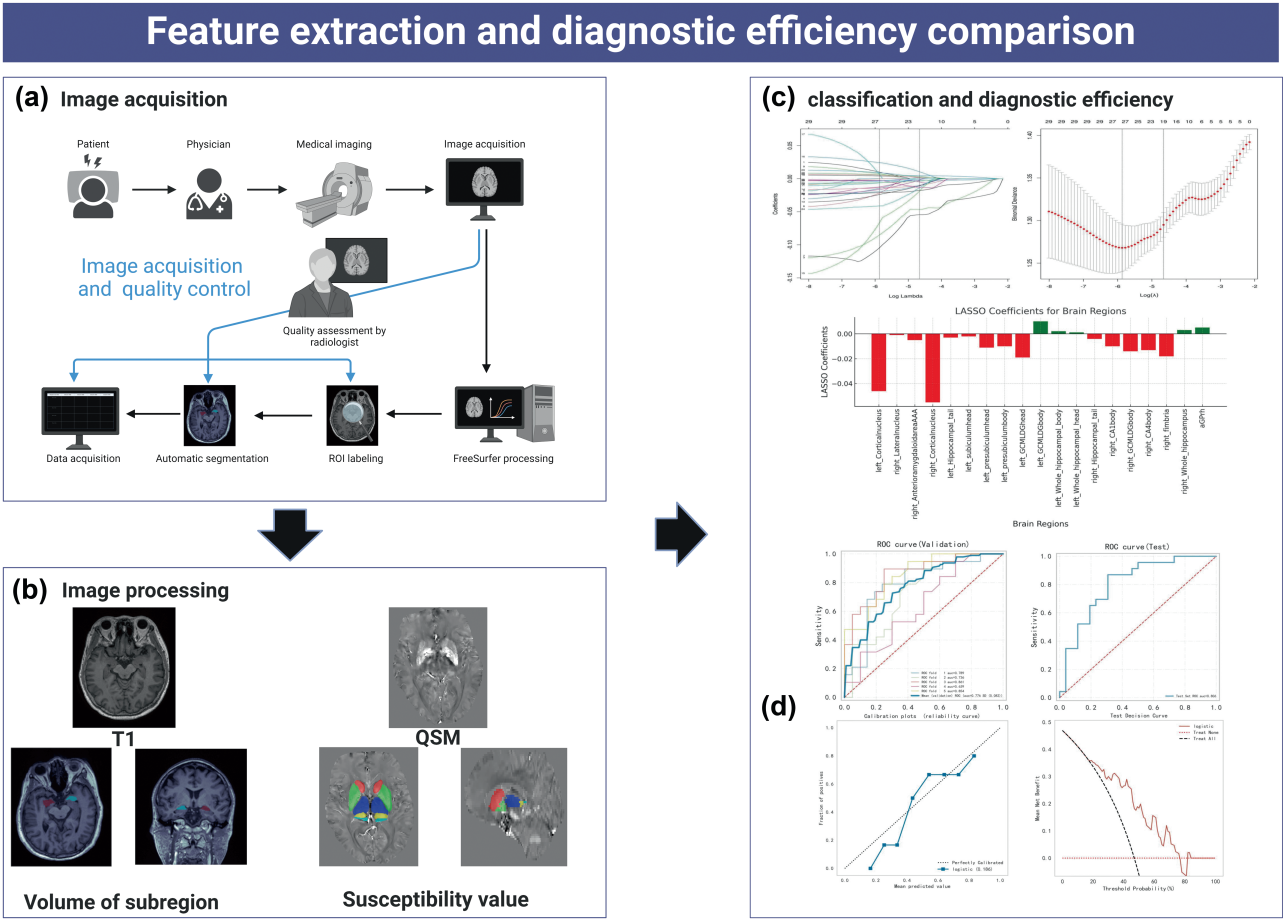
(a) Image acquisition and calibration process. (b) Image analyses were performed using the FreeSurfer image analysis suite implemented by fMRIPrep 20.1.1, followed by the toolbox of segmentation of hippocampal subregions and amygdala. The QSM was calculated on the Matlab19 platform. (c) Lasso regression screening features and construction of radiomics model. Radiomics model = 2.931–0.046 × left‐Cortical‐nucleus − 0.001 × right‐Lateral‐nucleus − 0.005 × right‐Anterior‐amygdaloid‐area − 0.055 × right‐Cortical‐nucleus − 0.003 × left‐Hippocampal‐tail − 0.002 × left‐subiculum‐head − 0.011 × left‐presubiculum‐head − 0.01 × left‐presubiculum‐body − 0.019 × left‐GCMLDG‐head + 0.01 × left‐GCMLDG‐body + 0.002 × left‐Whole‐hippocampal‐body + 0.001 × left‐Whole‐hippocampal‐head − 0.004 × right‐Hippocampal‐tail − 0.01 × right‐CA1‐body − 0.014 × right‐GCMLDG‐body−0.013 × right‐CA4‐body − 0.018 × right‐fimbria + 0.003 × right‐Whole‐hippocampus + 0.005 × aGPrh. (d) Radiomics model performance curve in diagnosing CSVD. aGPrh, globus pallidus right hemisphere; CSVD, cerebral small vessel disease; HC, health control; LASSO, least absolute shrinkage and selection operator; multivariate logistic regression; QSM, quantitative sensitivity map; ROI, region of interest.

We established a binomial Lasso regression model using 10‐fold cross‐validation on a dataset of approximately 250 subjects. The model was configured with nlambda set to 100. Feature selection was based on minimizing the cross‐validation error, with a minimum distance standard error *λ* of 0.009 chosen for robust and reliable feature selection (Figure 2C).

### 2.5 Radiomics model construction and validation

We employed the logistic regression machine learning method for classification, with CSVD as the classification variable and radiomics score as the feature in the model. The model parameters were set as follows: regularization factor (C) at 1.0, maximum number of iterations (max_iter) at 100, type of regularization (penalty) as l2, and tolerance for stopping criteria (tol) at 0.0001. To ensure repeatability, we used a fixed random seed (44) for consistent data splitting across cross‐validation runs. The model was validated using fivefold cross‐validation, where the dataset was divided into five subsets. In each iteration, one subset was used as the validation set, and the remaining four subsets were used for training. This process was repeated five times, ensuring each subset served as the validation set once. This approach ensured robust validation and helped prevent overfitting, providing reliable and consistent results (Figure 2d).

### 2.6 Hybrid model construction and validation

The radiomics model was then combined with clinical cognitive and motor test results to construct a hybrid model. The construction of the hybrid model used logistic regression and the stepwise selection method. The test set was used to evaluate the effectiveness of the hybrid model in diagnosing CSVD and associated CI. After performing fivefold cross‐validation, the results from each fold were averaged to create the hybrid model (Figure S2). This involved aggregating the predictions or parameter estimates from each fold to generate a model that generalizes well across all subsets of the data. To further validate the final model, the entire dataset was split into a final training set and a test set, following an 80/20 split. Specifically, 80% of the data (196 subjects) was used to train the final model, and the remaining 20% of the data (49 subjects) was used to evaluate the hybrid model's performance on unseen data. By averaging the results from cross‐validation and then validating the hybrid model on a separate test set.

### 2.7 Feature contributions and CI

The TMT score is a key test for the differential diagnosis of CSVD. To study the correlation between TMT scores and changes in brain region features, partial least squares (PLS) regression was used to identify significant brain region features based on their contribution rates, with optimal features selected according to the explained variance by number of components. Hierarchical regression analysis was then performed to investigate the impact of the highest contributing brain regions on cognitive ability (TMT score) in both HC and CSVD groups.

## 3 RESULTS

### 3.1 The participant demographics

There were significant differences in age and years of education between CSVD group and HC group, but there was no significant difference in gender between the two groups. The scores of MoCA, AVLT, SDMT, SCWT, and other cognitive tests in CSVD group were significantly worse than those in HC group. The proportion of hypertension and diabetes in the CSVD group was also significantly higher than that in the HC group. There was no significant difference in the content of A*β*142, A*β*140, Tau protein, and other pathological proteins between the two groups (Table 1).

**TABLE 1:** Baseline data of the cohort subjects.

Demographics	Group	Total (*n* = 245)	HC (*n* = 127)	CSVD (*n* = 118)	*p*	
Age, median [IQR]		60.000 [53, 66]	57.000 [52, 63]	63.000 [58, 67]	<.001	Mann–Whitney *U*
Gender, *n* (%)	Male	128 (52.245)	61 (48.031)	67 (56.780)	.171	Chi‐square test
	Female	117 (47.755)	66 (51.969)	51 (43.220)		
Hypertension, *n* (%)	No	144 (58.607)	85 (66.142)	59 (50.427)	.013	Chi‐square test
	Yes	101 (41.393)	43 (33.858)	58 (49.573)		
Diabetes, *n* (%)	No	133 (54.545)	79 (62.205)	54 (46.087)	.012	Chi‐square test
	Yes	112 (45.455)	49 (37.795)	63 (53.913)		
Hyperlipidemia, *n* (%)	No	134 (54.936)	72 (57.500)	62 (52.212)	.418	Chi‐square test
	Yes	111 (45.064)	54 (42.500)	57 (47.788)		
Smoke, *n* (%)	No	184 (75.102)	97 (76.378)	87 (73.729)	.632	Chi‐square test
	Yes	61 (24.898)	30 (23.622)	31 (26.271)		
Drinking, *n* (%)	No	161 (65.714)	87 (68.504)	74 (62.712)	.340	Chi‐square test
	Yes	84 (34.286)	40 (31.496)	44 (37.288)		
BMI, median [IQR]		24.655 [22.600, 27.171]	24.655 [22.583, 26.841]	24.609 [23.121, 27.580]	.428	Mann–Whitney *U*
HbA1c, median [IQR]		6.500 [5.700, 8.300]	6.200 [5.600, 7.700]	6.700 [5.900, 8.900]	.014	Mann–Whitney *U*
Glu, median [IQR]		6.200 [5.390, 8.100]	6.090 [5.200, 7.840]	6.410 [5.660, 8.580]	.137	Mann–Whitney *U*
CHOL, mean (±SD)		5.033 ± 1.124	5.105 ± 1.023	4.956 ± 1.218	.353	*t*‐Test
HDL, median [IQR]		1.360 [1.130, 1.570]	1.380 [1.160, 1.630]	1.320 [1.100, 1.550]	.108	Mann–Whitney *U*
LDL, mean (±SD)		3.097 ± 0.846	3.112 ± 0.840	3.082 ± 0.851	.804	*t*‐Test
Education, median [IQR]		12.000 [9.000, 16.000]	15.000 [12.000, 16.000]	12.000 [9.000, 15.000]	<.001	Mann–Whitney *U*
MoCA, median [IQR]		25.000 [22.000, 27.000]	25.000 [23.000, 28.000]	24.000 [21.000, 26.000]	.001	Mann–Whitney *U*
AVLT, mean (±SD)		55.551 ± 13.408	57.709 ± 13.028	53.229 ± 13.422	.009	*t*‐Test
SDMT, mean (±SD)		34.408 ± 14.294	38.835 ± 13.472	29.644 ± 13.606	<.001	*t*‐Test
SCWT, median [IQR]		131.000 [110.000, 156.000]	122.000 [107.000, 149.000]	137.000 [118.000, 175.000]	.001	Mann–Whitney *U*
TMT, median [IQR]		216.000 [154.000, 309.000]	175.000 [144.000, 248.000]	259.000 [197.000, 380.000]	<.001	Mann–Whitney *U*
TUG, median [IQR]		8.910 [8.090, 9.820]	8.740 [7.780, 9.500]	9.230 [8.460, 10.110]	.001	Mann–Whitney *U*
A*β*142, mean (±SD)		167.371 ± 34.986	170.317 ± 35.462	163.617 ± 34.003	.223	*t*‐Test
A*β*140, median [IQR]		268.053 [203.323, 316.162]	264.068 [201.465, 304.068]	281.306 [210.662, 318.467]	.217	Mann–Whitney *U*
Total‐Tau, median [IQR]		138.351 [110.668, 175.812]	138.351 [113.230, 174.485]	138.688 [108.109, 175.812]	.499	Mann–Whitney *U*
PTau18, median [IQR]		288.365 [232.786, 342.847]	284.249 [222.545, 335.601]	298.691 [255.407, 348.373]	.113	Mann–Whitney *U*

### 3.2 Feature selection

In the Lasso regression coefficient profile (Figure S1), the changes in the regression coefficients of each variable at different lambda values are displayed. The Lasso regression cross‐validation curve shows the mean squared error of the model at different lambda values, with the lambda value corresponding to the minimum mean squared error being 0.024. Based on this lambda value, the final model selected the following variables: left‐Cortical‐nucleus, right‐Lateral‐nucleus, right‐Central‐nucleus, right‐Medial‐nucleus, right‐Cortical‐nucleus, left‐Hippocampal‐tail, left‐presubiculum‐head, left‐molecular‐layer‐HP‐head, left‐molecular‐layer‐HP‐body, left‐GCMLDG‐head, left‐CA3‐body, right‐Hippocampal‐tail, right‐CA1‐body, right‐molecular‐layer‐HP‐head, right‐CA4body, right‐fimbria, right‐HATA, pHIPrh, aGPrh, pHIPlh, and lAMYlh. In the Lasso coefficient table, these selected variables are shown with their respective regression coefficients in the model, where red bars indicate negative coefficients and green bars indicate positive coefficients.

### 3.3 Radiomics model building and performance testing

The logistic machine learning model was constructed using the radiomics scores derived from the Lasso regression coefficients (Table 2). During the model construction and performance testing process, we first analyzed the stability and accuracy of the model through cross‐validation. In the ROC curve of the validation set, the AUC values for the five cross‐validation folds were 0.790, 0.756, 0.861, 0.907, and 0.854, resulting in an average AUC of 0.770 (standard deviation of 0.082). The ROC curve of the test set shows an AUC value of 0.806, further validating the model's excellent performance on unseen data. The decision curve analysis demonstrates that the radiomics model provides a higher net benefit across most threshold ranges and offers a significant net benefit in practical clinical applications (Figure 2c).

**TABLE 2:** Lasso regression coefficient table.

Characteristics	Coef
(Intercept)	2.931
Left‐cortical‐nucleus	−0.046
Right‐lateral‐nucleus	−0.001
Right‐anterior‐amygdaloid‐area	−0.005
Right‐cortical‐nucleus	−0.055
Left‐hippocampal‐tail	−0.003
Left‐subiculum‐head	−0.002
Left‐presubiculum‐head	−0.011
Left‐presubiculum‐body	−0.01
Left‐GCMLDG‐head	−0.019
Left‐GCMLDG‐body	0.01
Left‐whole‐hippocampal‐body	0.002
Left‐whole‐hippocampal‐head	0.001
Right‐hippocampal‐tail	−0.004
Right‐CA1‐body	−0.01
Right‐GCMLDG‐body	−0.014
Right‐CA4‐body	−0.013
Right‐fimbria	−0.018
Right‐whole‐hippocampus	0.003
aGPrh	0.005

*Note*: Feature selection was based on minimizing the cross‐validation error, with a minimum distance standard error *λ* of 0.009 chosen for robust and reliable feature selection.

### 3.4 Hybrid model building and performance testing

**FIGURE 3:**
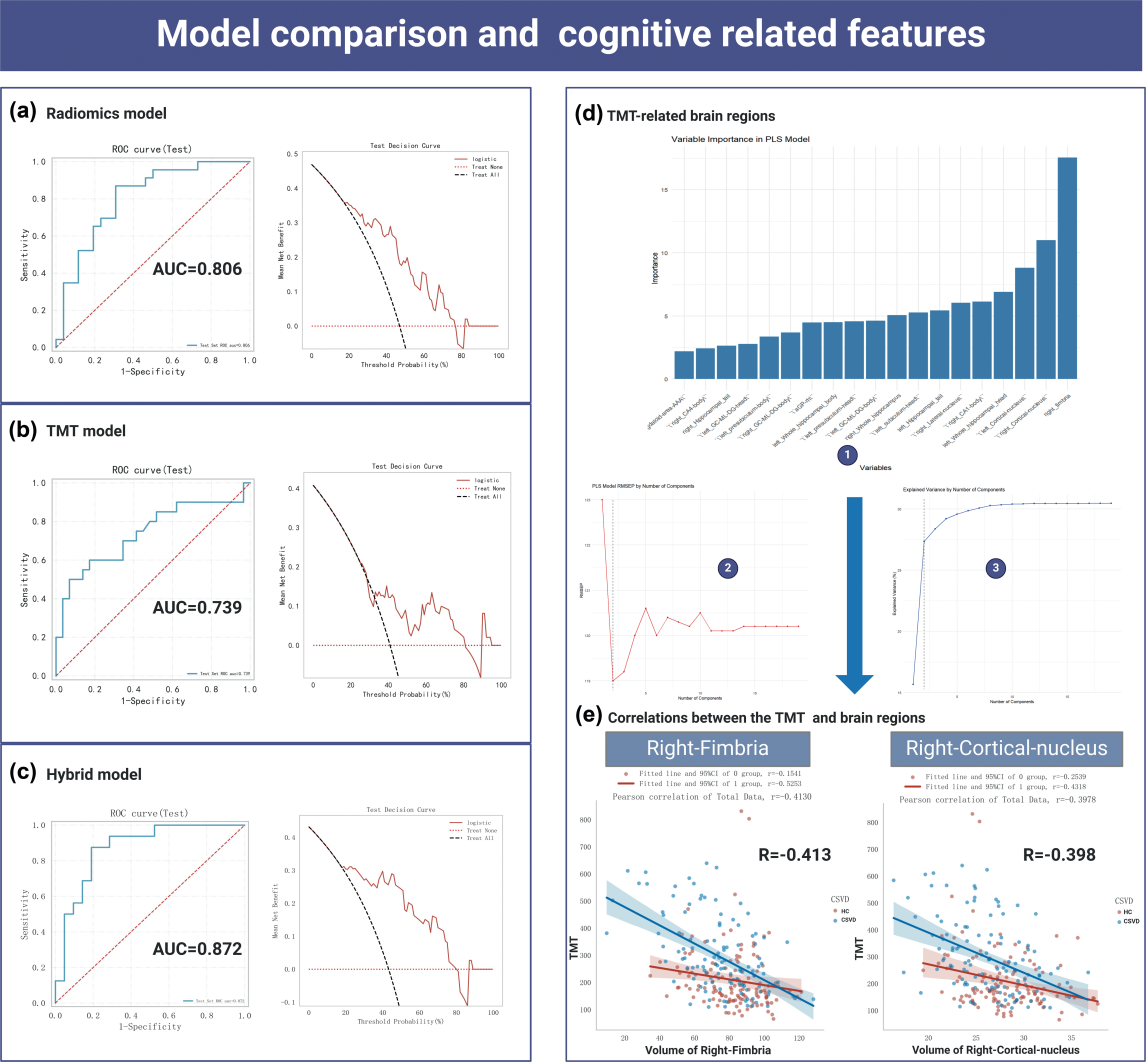
(a) ROC curve of radiomics model for diagnosing CSVD. (b) ROC curve of TMT model for diagnosing CSVD. The TMT model builds the model using only TMT tests and demonstrates its efficacy in CSVD diagnostics. (c) ROC curve of Hybrid model for diagnosing CSVD. Hybrid = 1.012 × radiomics + 0.003 × TMT + 1.012. (d1) The analysis using partial least squares (PLS) regression identified the most important brain regions related to the Trail Making Test (TMT) performance. The variable importance plot highlights the regions that significantly contribute to the predictive model. The right‐fimbria and right‐cortical‐nucleus emerged as the top predictors. (d2) The root mean squared error of prediction (RMSEP) by the number of components indicates that the model's performance stabilizes as the number of components increases, with no significant improvement beyond a certain point. This suggests an optimal number of components for the model, balancing complexity and predictive accuracy. (d3) The explained variance by the number of components shows that the proportion of variance explained by the model increases sharply with the initial components and levels off afterward. This pattern confirms that additional components beyond the optimal point add minimal explanatory power. (e) Hierarchical regression analysis of right‐fimbria and right‐cortical nucleus volume change and TMT performance. Right‐fimbria, hippocampus subregion right‐fimbria; right‐cortical‐nucleus: amygdala subregion right‐cortical‐nucleus.

Stepwise logistic regression was used for model construction, and finally, radiomics scores and TMT tests were incorporated into the hybrid model (Figure 3), with the formula: Hybrid = 1.012 × Radiomics + 0.003 × TMT + 1.012. Decision curve analysis demonstrates that across most threshold ranges, the hybrid model provides a higher net benefit compared to the radiomics model and the TMT model in diagnosing CSVD. Additionally, the ROC curve shows that the hybrid model achieved an AUC of 0.872, which is higher than the radiomics model (AUC = 0.806) and the TMT model (AUC = 0.739), further confirming its superior diagnostic performance (Figure 3).

### 3.5 Correlations between the TMT scores and radiomics features

The results of PLS regression indicated that the right fimbria and right cortical nucleus were the most important variables in the PLS model, significantly impacting TMT scores (Figure 3d). The RMSEP curve of the model showed that prediction errors stabilized as the number of components increased, while the explained variance curve revealed that the two components had the strongest explanatory power. The relationship between the right fimbria and TMT scores exhibited a Pearson correlation coefficient of −0.4130, indicating a negative correlation, which was stronger in the CSVD group (*r* = −.5253) than in the HC group (*r* = −.1541). Similarly, the right cortical nucleus and TMT scores had a Pearson correlation coefficient of −0.3978, also showing a negative correlation, stronger in the CSVD group (*r* = −.4318) than in the HC group (*r* = −.2539) (Figure 3e).

Moreover, hierarchical regression results (Table 3) further confirmed these findings. The right cortical nucleus showed a significant negative association with TMT scores (*β* = −13.404, *p* < .001). After adjusting for gender, years of education, history of diabetes, and hypertension, this association remained significant in both the total group (*β* = −11.972, *p* < .001) and the CSVD group (*β* = −15.242, *p* < .001), but not in the HC group (*β* = −4.132, *p* = .075). For the right fimbria, a significant negative association was found (*β* = −2.817, *p* < .001), which persisted in the CSVD group (*β* = −3.364, *p* < .001) after adjustment, but was not significant in the HC group (*β* = 0.736, *p* = .179).

**TABLE 3:** Correlations between the TMT scores and brain region features.

	Hierarchical	*N*	*β*	95%CI	*p*‐Value	*N* (adjusted)	*β* (adjusted)	95%CI (adjusted)	*p*‐Value (adjusted)
Right‐cortical‐nucleus	Total	245	−13.404	[−17.291, −9.517]	.000	245	−11.972	[−15.761, −8.183]	.000
	HC	127	−7.784	[−12.984, −2.585]	.003	127	−4.132	[−8.686, 0.421]	.075
	CSVD	118	−14.917	[−20.587, −9.247]	.000	118	−15.242	[−20.994, −9.491]	.000
Right‐fimbria	Total	245	−2.817	[−3.599, −2.036]	.000	245	−2.264	[−3.075, −1.452]	.000
	HC	127	−1.057	[−2.245, 0.131]	.081	127	.736	[−0.338, 1.810]	.179
	CSVD	118	−3.364	[−4.356, −2.373]	.000	118	−3.562	[−4.576, −2.548]	.000

*Note*: Hierarchical regression results, adjusted: adjusted for age, gender*, years of education, history of diabetes, history of hypertension. *Although the gender difference between the CSVD group and the HC group was not significant, we still adjusted for gender as a confounding factor considering the previous literature has confirmed the influence of gender on CSVD.

## 4 DISCUSSION

CSVD is a significant risk factor for CI and dementia in middle‐aged and elderly people, which can only be detected by MRI combined with professional imaging diagnosis. The hybrid model used in this study to diagnose CSVD can be used in clinical practice and can be integrated into the treatment process. Our study had two aims: (a) to develop and test a radiomics diagnostic model for CSVD based on automatic segmentation techniques in CSVD patients and (b) to explore the crucial tests and related brain regions for improving the diagnostic efficiency of CSVD.

The AUC of our radiomics model based on subregion volume and subcortical nuclei susceptibility values was 0.79 in the test set. However, the hybrid model, combining the TMT test and radiomics score, performed best in diagnosing CSVD (AUC, 0.87) among all groups. Compared with the previous imaging diagnostic criteria, this hybrid model achieves quantitative diagnosis of CSVD, and the feature processing technology based on automatic segmentation further improves the diagnostic ability. Previous studies have confirmed that processing capacity decline is an important indicator of CSVD patients (Tap et al., 2023). Therefore, we innovatively incorporated cognitive tests into the model to achieve further improvement in diagnostic performance.

Among all the subregion volume changes, the left and right cortical nuclei contributed the most to the radiomics, which also indicated that the left and right cortical nuclei atrophy played an important role in the pathogenesis and progression of CSVD patients. Dysregulation of neuroplasticity in the amygdala has long been recognized as an important cause of cognitive deterioration (Wang et al., 2023). However, the effects of changes in the external environment on changes in amygdala function remain largely unknown. We speculate that changes in the brain environment caused by CSVD, such as blood flow changes and metabolic disorders, will lead to the occurrence of neuroinflammation, which directly or indirectly promotes amygdala atrophy (Zheng et al., 2021). Moreover, alterations in the structure of cortical subregions may related to changes in neural circuits, potentially arising as a result of the disease development and progression (Liao et al., 2024; Meisner et al., 2022).

Brain iron deposition is mainly found in dystrophic microglia, iron‐containing macrophages, and astrocytes, implying that the development of inflammation and blood–brain barrier disorders play an important role in iron accumulation (Dusek et al., 2022). QSM can qualitatively and quantitatively detect brain iron deposition. In the present study, patients with CSVD showed a marked increase in globus pallidus susceptibility values, which may be due to increased iron deposition in the striatum of patients with CSVD (Li, Nguyen, et al., 2022). Previous evidence from animal and histological studies has shown a correlation between increased hippocampal and thalamic iron and poorer memory, and between iron in the globus pallidus and general cognition (Spence et al., 2020). Iron deposition in globus pallidus may be one of the important causes of cognitive decline in patients with CSVD, and the specific mechanism still needs to be further studied. In addition, Zhou et al. found that lymphatic system dysfunction may be related to iron deposition in the brain. Their study showed that QSM values of caudate nucleus, putamen, pallidum, thalamus, red nucleus, substantia nigra, and dentate nucleus were all correlated with ALPS index, which also provides a reference for further exploration of the mechanism of iron deposition in CSVD (Zhou et al., 2020).

Standards for Reporting Vascular Changes on Neuroimaging, an authoritative guideline for the diagnosis of CSVD, has defined the indications for the diagnosis of CSVD and has become the recognized authoritative standard in the field of CSVD (Wardlaw et al., 2013). However, the above guidelines do not fully explain the clinical manifestations of CSVD, nor mention the cognition and lesion assessment of CSVD. CI is an important clinical manifestation of patients with CSVD. Cognitive decline not only manifests in the late stage of CSVD, but also may occur in the early stage of the disease. Among the selected cognitive tests covering attention, executive ability, control and other aspects, TMT test shows the best diagnostic efficiency for CSVD. The TMT test includes two subtables A and B. The TMT‐A mainly measures visuospatial ability and writing‐motor speed, and the TMT‐B mainly measures processing speed and cognitive flexibility. We combined Part A with Part B to comprehensively evaluate the processing speed and visual processing ability of patients. In a previous survey of 13,688 studies and included 298 in the qualitative data synthesis, the TMT emerged as the specifically chosen cognitive domain and neuropsychological tool for assessing CI associated with CSVD, The utilization rate of TMT in all specialized second‐level cognitive tests reached 32%, which was the second highest among all tests (Salvadori et al., 2021).

The results based on Hierarchical regression analysis showed that atrophy of the right cortical nucleus and the right fimbria was significantly associated with decreased processing ability in CSVD patients. Current studies have confirmed the important role of amygdale cortical nucleus atrophy in AD, but the role of its structural changes in CSVD is still unclear (Qu et al., 2023). Our study found that cortical nuclear atrophy of the right amygdala is often accompanied by a decline in processing ability, and this structural change may be one of the important reasons for the decline in processing ability in CSVD patients. The hippocampal fimbria is also a structure implicated in cognitive function and dementia (Evans et al., 2018; Li, Yue, et al., 2022). However, given its complex and encapsulated internal structure, however, its role in cognition and dementia risk remains relatively inadequate. Our results showed that the hippocampal fimbria of CSVD patients showed significant atrophy, and the volume atrophy was often accompanied by the decline of processing ability, and this pattern was particularly significant in CSVD patients, which may indicate that the changes in the brain microenvironment of CSVD patients promote the atrophy of the hippocampal fimbria, thereby accelerating the decline of processing ability.

This study has some limitations. First, it should be noted that the other metals we omitted may also have an effect on the signal strength, but they are very insignificant compared to iron. Second, we only selected the susceptibility values of the major subcortical nuclei, which may lead to limitations of the results. In addition, since this project is a prospective cohort study, all subregion segmentation and susceptibility value calculation are focused on specific regions, and the small number of features may have an impact on the generalization of the results. In the future, more characteristics should be included and repeated studies should be conducted to demonstrate the robustness of this study.

## FUNDING INFORMATION

This study was supported by the Natural Science Foundation of Shandong Province (grant number ZR2020MH288), Study Abroad Program by Shandong Province (grant number 201803059), Technology Development Plan of Jinan (grant number 202328066), Shandong Provincial Medical and Health Science and Technology Development Plan (grant numbers 202309010557 and 202309010560), and Shandong Province Medical System Employee Science and Technology Innovation Plan (grant number SDYWZGKCJH2023034).

## CONFLICT OF INTEREST STATEMENT

The authors declare that there are no conflicts of interest.

## Supporting information


**FIGURE S1:** Lasso regression model. The Lasso regression model was established by binomial, and the standard error *λ* of the minimum distance was 0.009 for screening, the selected variables included 18 subregion volumes and a cortical magnetic susceptibility value.
**FIGURE S2:** Construction of the Hybrid model based on fivefold cross‐validation.
**FIGURE S2:** The ROC AUC values for each fold on the validation set are as follows: ROC fold 1: AUC = 0.844, ROC fold 2: AUC = 0.861, ROC fold 3: AUC = 0.795, ROC fold 4: AUC = 0.804, ROC fold 5: AUC = 0.691.

## Data Availability

Research data are not shared.
