# Supplementary material for: Advancing cerebral small vessel disease diagnosis: Integrating quantitative susceptibility mapping with MRI‐based radiomics
Source: Hum Brain Mapp. 2024 Sep 10;45(13):e70022. doi: 10.1002/hbm.70022 (PMC11386328; doi:10.1002/hbm.70022)
Supplement: Supplementary file 1 — FIGURE S1: Lasso regression model. The Lasso regression model was established by binomial, and the standard error λ of the minimum distance was 0.009 for screening, the selected variables included 18 subregion volumes and a cortical magnetic susceptibility value. FIGURE S2: Construction of the Hybrid model based on fivefold cross‐validation. FIGURE S2: The ROC AUC values for each fold on the validation set are as follows: ROC fold 1: AUC = 0.844, ROC fold 2: AUC = 0.861, ROC fold 3: AUC = 0.795, ROC fold 4: AUC = 0.804, ROC fold 5: AUC = 0.691. [file HBM-45-e70022-s001.docx]

**Figure S1 : Lasso regression model**


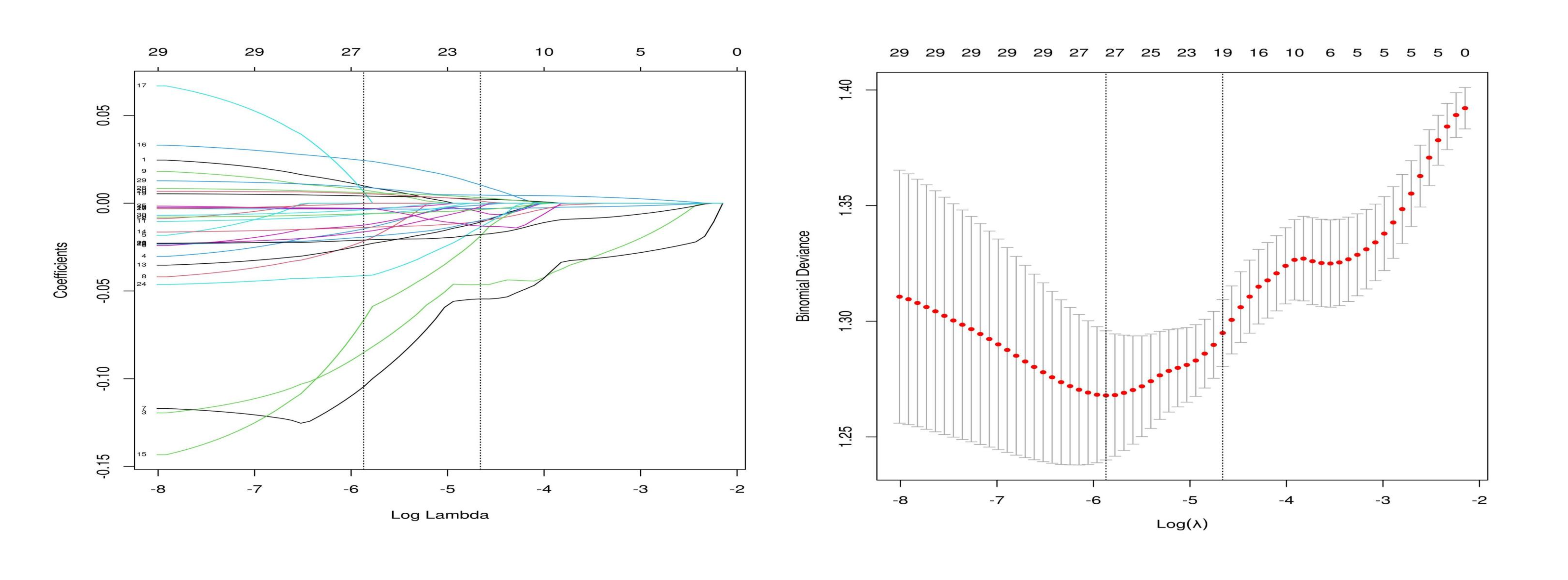


**Figure S1 :** The Lasso regression model was established by binomial, and the standard error λ of the minimum distance was 0.009 for screening, the selected variables included 18 subregion volumes and a cortical magnetic susceptibility value.

**Figure S2 : Construction of the Hybrid model based on 5-fold cross-validation**


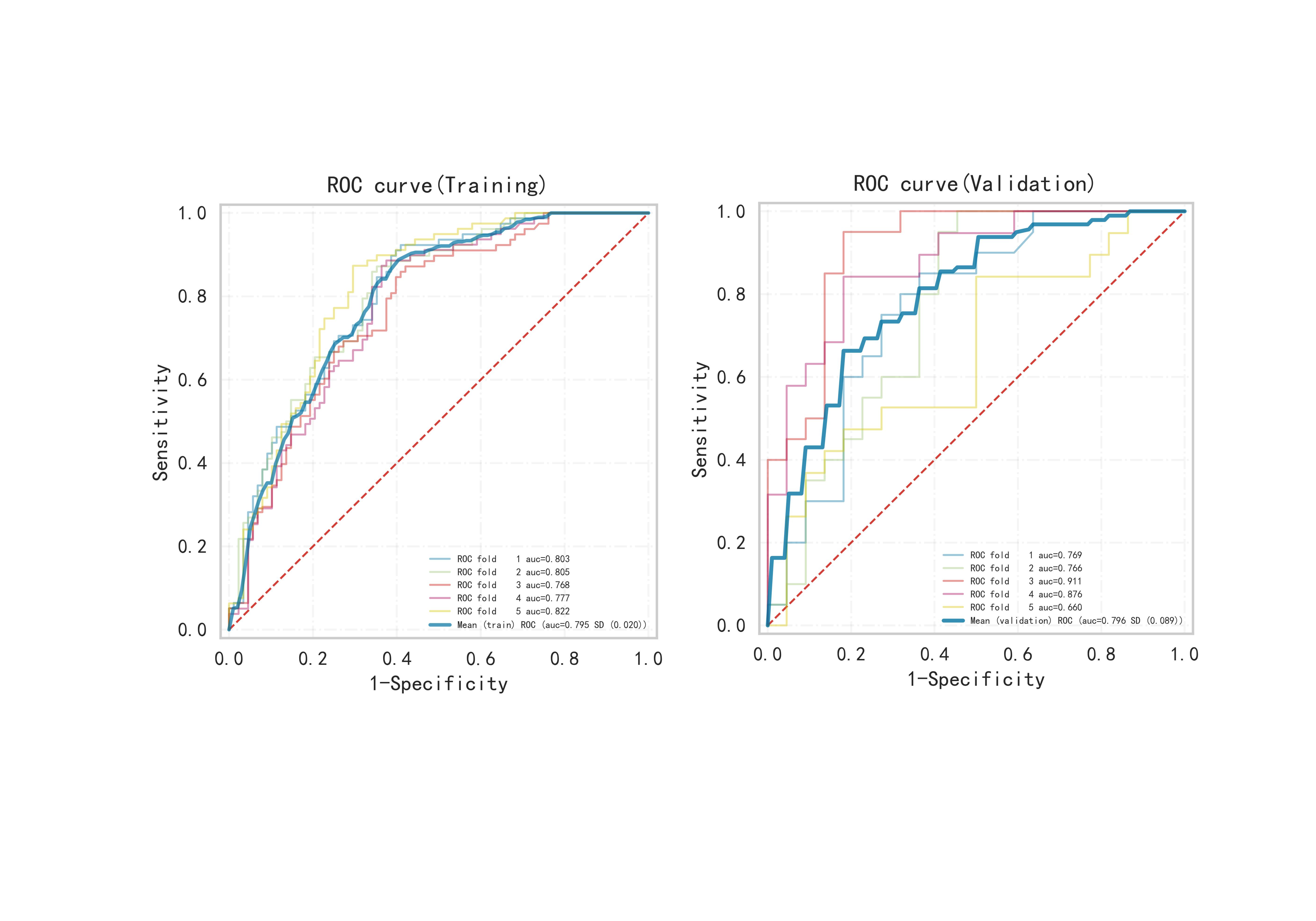


**Figure S2 :** The ROC AUC values for each fold on the validation set are as follows: ROC fold 1: AUC = 0.844, ROC fold 2: AUC = 0.861, ROC fold 3: AUC = 0.795, ROC fold 4: AUC = 0.804, ROC fold 5: AUC = 0.691.
